# Bringing Packed Red Blood Cells to the Point of Combat Injury: Are We There Yet?

**DOI:** 10.4274/tjh.2018.0081

**Published:** 2018-08-05

**Authors:** Aytekin Ünlü, Soner Yılmaz, Özlem Yalçın, Metin Uyanık, Patrizio Petrone, Rıza Aytaç Çetinkaya, İbrahim Eker, Murat Urkan, Taner Özgürtaş, İsmail Yaşar Avcı, Nazif Zeybek, Ali Cenk Aksu

**Affiliations:** 1 University of Health Sciences, Gülhane Training and Research Hospital, Clinic of General Surgery, Ankara, Turkey; 2 University of Health Sciences, Gülhane Training and Research Hospital, Regional Blood and Training Center, Ankara, Turkey; 3 Koç University Faculty of Medicine, Laboratory of Hemorheology, Hemodynamics, and Vascular Biology, İstanbul, Turkey; 4 Çorlu State Hospital, Clinic of Biochemistry, Tekirdağ, Turkey; 5 NYU Winthrop Hospital, Clinic of Surgery, Mineola; New York Medical College, Valhalla, New York, USA; University of Las Palmas de Gran Canaria, Canary Islands, Spain; 6 University of Health Sciences, Sultan Abdulhamid Han Training and Research Hospital, Clinic of Infectious Diseases, İstanbul, Turkey; 7 Afyon Kocatepe University Faculty of Medicine, Department of Pediatric Hematology, Afyonkarahisar, Turkey; 8 University of Health Sciences, Gülhane Training and Research Hospital, Clinic of Biochemistry, Ankara, Turkey; 9 University of Health Sciences, Gülhane Training and Research Hospital, Clinic of Infectious Diseases and Clinical Microbiology, Ankara, Turkey

**Keywords:** Combat trauma, Blood transport, Prehospital transfusion, Hemolysis

## Abstract

**Objective:**

Hemorrhage is the leading cause of injury-related prehospital mortality. We investigated worst-case scenarios and possible requirements of the Turkish military. As we plan to use blood resources during casualty transport, the impact of transport-related mechanical stress on packed red blood cells (PRBCs) was analyzed.

**Materials and Methods:**

The in vitro experiment was performed in the environmental test laboratories of ASELSAN^®^. Operational vibrations of potential casualty transport mediums such as Sikorsky helicopters, Kirpi^®^ armored vehicles, and the NATO vibration standard MIL-STD-810G software program were recorded. The most powerful mechanical stress, which was created by the NATO standard, was applied to 15 units of fresh (≤7 days) and 10 units of old (>7 days) PRBCs in a blood cooler box. The vibrations were simulated with a TDS v895 Medium-Force Shaker Device. On-site blood samples were analyzed at 0, 6, and 24 h for biochemical and biomechanical analyses.

**Results:**

The mean (±standard deviation) age of fresh and old PRBCs was 4.9±2.2 and 32.8±11.8 days, respectively. Six-hour mechanical damage of fresh PRBCs was demonstrated by increased erythrocyte fragmentation rates (p=0.015), hemolysis rates (p=0.003), and supernatant potassium levels (p=0.003) and decreased hematocrit levels (p=0.015). Old PRBC hemolysis rates (p=0.015), supernatant potassium levels (p=0.015), and supernatant hemoglobin (p=0.015) were increased and hematocrit levels were decreased (p=0.015) within 6 h. Two (13%) units of fresh PRBCs and none of the old PRBCs were eligible for transfusion after 6 h of mechanical stress.

**Conclusion:**

When an austere combat environment was simulated for 24 h, fresh and old PRBC hemolysis rates were above the quality criteria. Currently, the technology to overcome this mechanical damage does not seem to exist. In light of the above data, a new national project is being performed.

## Abstract (tr)

**Amaç:**

Kan kayıpları, hastane öncesi dönemdeki yaralanmalara bağlı ölümlerin en sık sebebidir. Türk ordusu için en kötü senaryoları ve olası ihtiyaçları araştırdık. Çatışma alanından nakil esnasında kan kaynaklarını kullanmayı planladığımız için nakil işleminden kaynaklı mekanik stresin eritrosit konsantreleri üzerine etkisini analiz edilmiştir.

**Gereç ve Yöntemler:**

İn vitro çalışmalar ASELSAN^®^’ın dış ortam test laboratuvarlarında gerçekleştirildi. Çatışma alanında kan taşıma işleminde kullanılması muhtemel olan Sikorsky helikopteri ve Kirpi^®^ araçlarının operasyonel vibrasyonları ve NATO MIL-STD-810G titreşim standart yazılımı kayıt altına alındı. NATO standardının en güçlü titreşime neden olduğu hesaplandı. Kan saklama çantası içindeki 15 ünite taze (≤7 gün) ve 10 ünite taze olmayan eritrosit konsantresi (>7 gün), NATO standardı olan mekanik strese maruz bırakıldı. Titreşim TDS v895 Medium-Force Shaker cihazı tarafından simüle edildi. Simülasyonun 0., 6. ve 24. saatinde biyokimyasal ve biyomekanik analiz için kan örnekleri alındı.

**Bulgular:**

Taze ve taze olmayan eritrosit konsantreleri sırasıyla ortalama 4,9 [standart deviasyon (SD) ±2,2] ve 32,8 (SD ±11,8) günlüktü. Taze eritrosit konsantrelerinde 6. saatte gelişen mekanik hasar; artmış eritrosit fragmentasyonu (p=0,015), hemoliz oranı (p=0,003) ve supernatant potasyum (p=0,015) düzeyleri ile gösterildi. Taze olmayan eritrosit konsantrelerinin 6. saatte hemoliz oranı (p=0,015) ve supernatant potasyum düzeyi (p=0,015) yükselirken, hematokrit değerleri (p=0,015) düştü. İlk 6 saat içerisinde taze eritrosit konsantrelerinin 2’si (%13) transfüze edilebilir kalitede kalırken, taze olmayanların ise hiçbirisi uygun değildi.

**Sonuç:**

Taze ve taze olmayan eritrosit konsantrelerinin hemoliz oranları, bu ürünleri muharebe sahasında taşıdığımızda, çoğunun kullanılamaz hale geleceğini göstermektedir. Halihazırda eritrositler üzerinde oluşan mekanik hasarlanmanın önüne geçebilecek bir teknoloji bulunmamaktadır. Yukarıdaki verilerin ışığı altında yeni bir ulusal proje gerçekleştirilmektedir.

## Introduction

During the last century, 90% of combat-related deaths occurred in the prehospital period (PHP), which only decreased to 75%-87% in recent military conflicts [1,2,3,4,5,6,7]. PHP mortality may be stratified as nonsurvivable (75%) and potentially survivable (25%) from a medical perspective [5,6,7]. The majority of potentially survivable PHP deaths (90%) were attributed to hemorrhage [7].

The Department of War Surgery of the University of Health Sciences has regarded the above data from recent military conflicts as an essential field of medical research. In order to decrease preventable injury-related deaths in the PHP, acquiring the capability of en route blood transfusion by transporting packed red blood cells (PRBCs) to the point of injury has proved valuable [8].

In a worst-case scenario, prolonged transportation of the already limited PRBC resources by army tactical ambulances and helicopters may be required for casualty evacuation missions. However, movement of these vehicles creates mechanical vibrations with different amplitudes and frequencies, which exert mechanical stress on PRBCs. We investigated the biochemical and biomechanical parameters of PRBCs exposed to vibration for 24 h.

## Materials and Methods

The study was designed as a within-subjects, in vitro experiment and was approved by the Yeditepe University Clinical Research Ethics Committee (24/11/2016-682). The study was conducted at the Environmental Test Laboratory of ASELSAN Company (Macunköy, Ankara, Turkey).

### Preparation of PRBCs

All consenting volunteers that met the criteria of the 2016 Turkish National Blood and Blood Products Guide for blood donations were eligible for the study. PRBCs were prepared by the Gülhane Regional Blood and Training Center from whole blood as described by Cetinkaya et al. [9]. The mean volume of PRBCs was 220±40 mL. We refer to blood of ≤7 days as fresh and blood of >7 days as old PRBCs. The mean age of fresh and old PRBCs was 4.9 (SD: ±2.19) and 32.8 (SD: ±11.8), respectively. All PRBCs were placed in an MT-25E cooler box and transported to ASELSAN Laboratories in 15 min. A Tinytag View 2 (TV-4510, UK) temperature data logger was placed in the cooler box and records showed that the temperature remained between 3.2 °C and 3.9 °C.

### Vibration Analysis and Simulation of Vibration

The PRBCs were exposed to 24 h of simulated shaking by the LDS v895 Medium-Force Shaker Device (Brüel & Kjær Inc., Denmark). The first step was recording vibration profiles of potential PRBC carriers such as Sikorsky Blackhawk helicopters (S70) (routine flight) (Lockheed Martin, USA) and Kirpi multipurpose armored vehicles (rough terrain, 30 km/h speed) (BMC Inc., Turkey). Vibrations were recorded by the G-Sensor Pro v3.0.5 application for Android devices. Currently, the durability of all military equipment against vibrations is tested using the Test Method 514.6 (MIL-STD-810G, US Army Test and Evaluation Command, 2008) software program. Vibrations of Test Method 514.6 were also recorded. Root mean square acceleration values of three different vibration environments were calculated. Test Method 514.6 had the highest value and was chosen for testing PRBCs for 24 h (Table 1).

### Blood Sample Analyses

Hematocrit, pH, supernatant hemoglobin, supernatant osmolality, supernatant potassium, 2,3-diphosphoglycerate (2,3-DPG), ATP (adenosine 5′-triphosphate), osmotic fragility, erythrocyte deformability, and erythrocyte fragmentation were measured at 0, 6, and 24 h. One milliliter of blood was collected and immediately analyzed for hematocrit and pH measurements via the IRMA TruPoint Blood Gas Analyzer (ITC, System Version 7.1, USA). Supernatant osmolality, potassium (mmol/L), and hemoglobin (g/dL) were measured using a Radiometer ABL 800 (Radiometer Trading, Denmark). ATP was assayed using an ATP assay kit (ab83366; Abcam, UK) and 2,3-DPG was assayed using a human 2,3-DPG enzyme-linked immunosorbent assay kit (CK-E11265, Eastbiopharm, China). Osmotic fragility was calculated using the Parpart method [10]. Presence of hemolysis at 0.45%-0.55%, >0.55%, and ≤0.30% NaCl concentrations was defined as normal, increased, and decreased osmotic fragility, respectively. Supernatant hemoglobin values were measured using Drabkin’s method [11]. Erythrocyte deformability was determined using a laser-assisted optical rotational cell analyzer (LORCA; RR Mechatronics, the Netherlands). Elongation index (EI) was calculated during the application of 10 steps of shear stress (SS) in the range of 0.3 to 50 Pa; RBC deformability was expressed as EI-SS curves. These EI-SS data were characterized by the maximum EI at infinite SS (EI_max_) and the SS needed to achieve one-half of this maximum (SS_1/2_); the SS_1/2_/EI_max_ ratio was calculated as a normalized measure of SS_1/2_. SS_1/2_/EI_max_ is inversely related to RBC deformability such that a lower value indicates better deformability. Red blood cell fragmentation was determined using a Multisizer 3 cell counter system (Beckman Coulter, USA).

### Statistical Analysis

In order to determine the number of fresh and old PRBC units, we conducted a priori power analysis. PRBCs in a blood cooler box were exposed to Test Method 514.6 vibrations for 6 h and tested for hemolysis percent and potassium levels. Sample size analysis was performed using the Güç Analizi (Power Analysis) application (Savante Mobile Apps, Google Play). Sample size power was set at 80%. Analysis showed that 15 units of fresh and 10 units of old PRBCs were required for the study. All data were analyzed using SPSS 22 (IBM Corp., Armonk, NY, USA). The Friedman test was used to analyze differences within each group. Upon finding a statistically significant difference, analyses between comparison groups were performed using the Bonferroni-corrected Wilcoxon signed-ranks test. The Mann-Whitney U test was performed for analyzing the differences in biochemical and biomechanical values between the fresh and old PRBCs. As Friedman and Wilcoxon tests were performed for statistical analyses, we used median (minimum/maximum) values for comparative analyses and descriptive purposes. The level of statistical significance was set at 0.05.

## Results

Analyses between 0 and 6, 6 and 24, and 0 and 24 h were defined as comparisons 1, 2, and 3, respectively.

### Analysis of Fresh PRBCs

**Figure 1:**
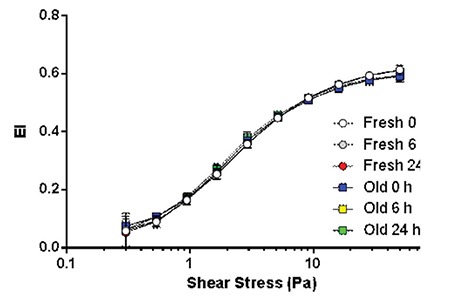
Elongation index of fresh and old samples measured at shear stresses between 0.3 and 50 Pa.

**Figure 2:**
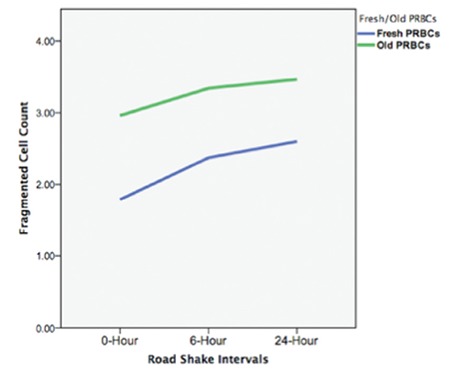
Cell counts between 15 and 40 fL (decreased cell volume due to fragmentation) of fresh and old samples measured with a Multisizer 3 (Beckman & Coulter, USA) (209x296 mm, 72x72 DPI). PRBCs: Packed red blood cells.

**Figure 3:**
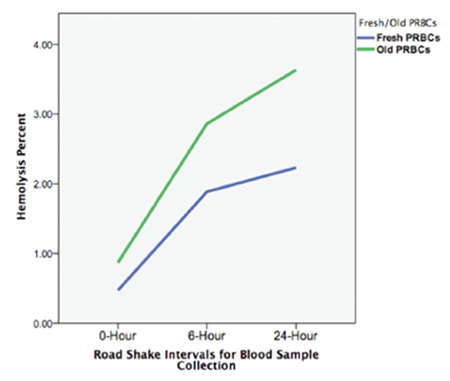
Differences in hemolysis percentages between fresh and old packed red blood cells during road shaking simulation. PRBCs: Packed red blood cells.

There were no statistically significant differences in the erythrocyte deformability parameters of the EI_max_ and SS_1/2_ values (p=0.14 and p=0.36, respectively) (Figure 1). However, statistically significant erythrocyte fragmentation occurred in comparison 1 [1.72 (1.13/2.43 vs. 2.29 (1.36/3.15), p=0.015], which continued to increase without statistical significance in comparison 2 [2.29 (1.36/3.15) vs. 2.24 (1.69/4.96), p=0.09] (Figure 2). Similarly, hemolysis of erythrocytes was statistically significantly increased in comparison 1 [0.37 (0.19/0.80) vs. 1.49 (0.47/5.09), p=0.003], which continued to increase in comparison 2 with borderline statistical significance [1.49 (0.47/5.09) vs. 1.74 (0.78/5.21), p=0.04] (Table 2). Unsurprisingly, the hemolysis percentage was also statistically significantly increased in comparison 3 [0.37 (0.19/0.80) vs. 1.74 (0.78/5.21), p=0.003] (Figure 3). Only two fresh PRBC units at 6 h and one PRBC unit at 24 h had hemolysis percentages less than 0.8%.

Simulated SS for 24 h was found to statistically significantly affect pH levels (p=0.001) (Table 2). However, the statistical significance was due to differences in comparison 1 [6.9 (6.9/7.08) vs. 6.8 (6.8/7.0), p=0.003], whereas comparisons 2 [6.8 (6.8/7.0 vs. 6.8 (6.7/7.1), p=1) and 3 [6.9 (6.9/7.08) vs. 6.8(6.7/7.1), p=0.05] were not statistically significantly different.

ATP levels decreased significantly in comparison 1 [90.2 (60.88/189.8) vs. 70.4 (38.2/102.9), p=0.003] and comparison 2 [70.4 (38.2/102.9) vs. 84.6 (63.9/166.8), p=0.04]. Likewise, 2,3-DPG levels also decreased statistically significantly in comparison 1 [1.06 (0.9/1.9) vs. 0.86 (0.65/1.35), p=0.003], comparison 2 [0.86 (0.65/1.35) vs. 0.64 (0.53/0.84), p=0.003], and comparison 3 [1.06 (0.9/1.9) vs. 0.64 (0.53/0.84), p=0.003]. However, despite the statistically insignificant increase in osmotic fragility in comparison 1 [0.4 (0.35/0.40) vs. 0.40 (0.4/0.45), p=0.25], continued mechanical agitation resulted in a statistically significant increase in comparison 2 [0.40 (0.4/0.45) vs. 0.45 (0.4/0.45), p<0.001] and comparison 3 [0.4 (0.35/0.40) vs. 0.45 (0.4/0.45), p<0.001]. The supernatant hemoglobin levels were not significantly increased in comparison 1 [0.08 (0.02/0.17) vs. 0.07 (0.02/0.17), p=0.08] and comparison 2 [0.07 (0.02/0.17) vs. 0.11 (0.02/0.3), p=0.09]. However, the difference was statistically significant in comparison 3 [0.08 (0.02/0.17) vs. 0.11 (0.02/0.3), p=0.009]. Supernatant osmolality was statistically significantly decreased in comparison 1 [284 (248/307) vs. 265.4 (234/292), p=0.003], comparison 2 [265.4 (234/292) vs. 259 (227.7/293), p=0.003], and comparison 3 [284 (248/307) vs. 259 (227.7/293), p=0.003]. Naturally, mechanical agitation caused a significant increase in supernatant potassium in comparison 1 [21.8 (10.2/33.12) vs. 32.9 (21/40.7), p=0.003], comparison 2 [32.9 (21/40.7) vs. 37.7 (27/44.5), p=0.003], and comparison 3 [21.8 (10.2/33.12) vs. 37.7 (27/44.5), p=0.003]. As a result of fresh PRBC hemolysis, hematocrit levels were significantly decreased in comparison 1 [62.4 (50/79.1) vs. 48 (38.3/80), p=0.015] and comparison 3 [62.4 (50/79.1) vs. 46.4 (36/60.4), p=0.015) (Table 2).

### Analysis of Old PRBCs

No statistically significant differences in EI_max_ and SS_1/2_ values were found in any of the comparisons (p>0.05) (Figure 1). Fragmentation rates of old PRBCs continued to increase without significance in comparison 1 [3.01 (1.3/4.39) vs. 2.53 (1.62/5.69), p>0.05] and comparison 2 [2.53 (1.62/5.69) vs. 3.20 (1.3/6.71), p>0.05] (Figure 2). The median (min/max) hemolysis percentage of old PRBCs at 0, 6, and 24 h were 0.7 (0.26/0.8), 2.47 (1/6.44), and 2.95 (1.31/10.3), respectively, and the increases were statistically significant (p<0.05) (Table 2). Hemolysis percentages of all blood bags were above 0.8% after 6 h of simulation.

ATP and osmotic fragility values showed no significant changes in any of the comparisons (p>0.05). However, there were significant differences in supernatant osmolality values in comparison 1 [240.3 (191/274.1) vs. 235 (189.4/268), p=0.015], comparison 2 [235 (189.4/268) vs. 233.9 (185/266), p=0.015], and comparison 3 [240.3 (191/274.1) vs. 233.9 (185/266), p=0.015]. Supernatant potassium values in comparison 1 [38.6 (28.3/48.6) vs. 42.8 (32.9/53.5), p=0.015], comparison 2 [42.8 (32.9/53.5) vs. 43.5 (33.5/56.2)], and comparison 3 [38.6 (28.3/48.6) vs. 43.5 (33.5/56.2), p=0.015] were also statistically significant. Likewise, 2,3-DPG values in comparison 1 [1.16 (0.84/1.58) vs. 0.74 (0.6/1), p=0.015], comparison 2 [0.74 (0.6/1) vs. 0.61 (0.53/0.76), p=0.015], and comparison 3 [1.16 (0.84/1.58) vs. 0.61 (0.53/0.76), p=0.015] were statistically significantly different. Supernatant Hb levels increased significantly in comparison 2 [0.07 (0.02/0.11) vs. 0.19 (0.12/0.44), p=0.013], while differences between comparisons 1 [0.10 (0.03/0.2) vs. 0.07 (0.02/0.11), p=0.2] and 3 [0.10 (0.03/0.2) vs. 0.19 (0.12/0.44), p=0.06] were statistically insignificant. The hematocrit levels were also statistically significantly decreased in comparison 1 [58 (44.7/70.2) vs. 45.9 (34.3/56.6), p=0.015] and comparison 3 [58 (44.7/70.2) vs. 43.6 (34/49.5), p=0.015] (Table 2).

### Analyses of Differences between Fresh and Old PRBCs

2,3-DPG levels between the fresh and old PRBCs were not statistically significantly different (p>0.05). The ATP levels of fresh PRBCs at 0 h were significantly higher (p<0.001), but the differences in analysis at 6 and 24 h were not statistically significant (p>0.05). Supernatant hemoglobin levels and hemolysis percentages between fresh and old PRBCs were not statistically significantly different (p>0.05). Supernatant K levels of old PRBCs were statistically significantly higher than those of the fresh PRBCs in all three successive analyses (p<0.001, p<0.001, p=0.005, respectively). The other biochemical analyses are shown in Table 2. EI_max_ (p=0.003, p=0.004, p=0.004, respectively) and SS_1/2_ (p=0.031, p=0.036, p=0.004, respectively) values of fresh PRBCs were significantly better in all comparisons. Fragmentation rates of fresh erythrocytes were significantly lower at 0 h; however, the difference was not significant at 6 or 24 h (Table 2).

## Discussion

In order to prevent hemorrhage-related early mortality, strategies that comprise crystalloid use, a high ratio of fresh frozen plasma, and platelets in PRBC transfusion protocols have been developed[12]. Malsby et al. [13] reported the initial military experience of en route blood product transfusion for combat trauma casualties. Transfusions were started aboard upon receiving the casualties from the point of injury. More interestingly, clinical indications for transfusion were appreciated and executed by well-trained flight medics. They concluded that flight medic-initiated transfusions were safe and effective and studies to determine the effect of PHP transfusion on outcomes were required. Brown et al. [8]performed an outcomes study and showed that PHP PRBC transfusion was associated with significant 24-h and 30-day reduction in mortality rates. They also showed that trauma-induced coagulopathy was reduced by 88%.

In order to take the transfusion capability closer to the point of injury, some practical questions about blood logistics need answers for future planning, such as: “If PRBCs could be kept at 4 °C, would their quality be maintained after prolonged transport times?” Otani et al. [14] investigated whether a helicopter flight affected the quality and shelf-life of RBCs. Seven days after donation, five units of PRBCs were packed into a blood cooler box and transported in a helicopter for 4 h. Then they were stored again and their quality was evaluated 7, 14, 21, and 42 days after donation. Only supernatant hemoglobin and hemolysis levels were slightly increased 42 days after donation. Supernatant potassium, hematocrit, pH, and 2,3-DPG levels at 42 days remained unchanged.

Boscarino et al.[15] exposed 20 units of pooled PRBCs (7 days old) in a Golden Hour container to a 30,000-foot parachute descent, followed by carrying the container in a rucksack for 12 h in an environment of 48 °C and 9% humidity. They investigated the biochemical (pH, lactate, potassium, and ATP) and biomechanical (EI_max_, half EI_max_, percent hemolysis, and morphology) parameters and found no significant impact on markers of RBC stress.

In our worst-case scenario, blood supplies are limited and the forward-deployed fresh and old blood products will be subject to continuous shear forces due to perpetual tactical evacuation missions. As the above-mentioned studies’ simulated conditions showed no resemblance to our envisioned combat environment, we have designed a 24-h simulation study.

Our study was performed in “within limits” temperature settings, as hemolysis would increase linearly with temperature [16]. When blood is collected in a bag with limited amounts of dextrose, phosphate, and adenine to maintain ATP and 2,3-DPG levels, erythrocytes metabolize these preservatives to maintain their integrity. The lactate level increases progressively in the blood bag, which decreases the pH and 2,3-DPG levels during the storage period[17,18]. Decreased ATP levels reduce the deformability of the cells and cellular homeostasis [19]. Approximately 25% of ATP content and over 90% of 2,3-DPG is lost in a unit of PRBCs after 42 days of storage [20]. As this study lacks control groups, the decrease in erythrocyte metabolism-related parameters cannot be solely attributed to shear stress. The pH levels of fresh PRBCs decreased significantly in the first 6 h.

Storage temperatures of 1 °C to 4 °C slow the RBC metabolism and decrease the energy demand. However, storage at 4 °C impairs the ATP-dependent potassium pump, resulting in potassium leakage. The extracellular potassium concentration increases by approximately 1 mEq/L per day until the intracellular and extracellular potassium ions reach equilibrium. Potassium loading may be of clinical importance in patients receiving massive transfusions[21]. After 24 h, the supernatant potassium level of fresh and old PRBCs was significantly higher by 71% and 87% than the expected value.

According to the European Directorate for the Quality of Medicine-Healthcare of the Council of Europe (EDQM) criteria and the North American Blood Quality Licensure, the acceptable level of hemolysis has been set at 0.8% and 1%, respectively [22]. The EDQM hemolysis criterion has been approved by the Turkish National Blood and Blood Products Guide (2016)[23]. After 6 and 24 h of shear stress, only 2 (13%) and 1 (6.6%) of the fresh blood packs were eligible for transfusion, respectively. None of the old PRBCs were found eligible at the 6-h test. Mechanical agitation significantly increased hemolysis and fragmentation values of fresh and old PRBCs at 6-h and 24-h analyses. Decreases in hematocrit levels were observed in fresh and old PRBCs at the 6-h analysis.

Storage-induced red blood cell damage increases osmotic fragility, especially after 5 weeks [24]. Increased osmotic fragility was evident in old PRBCs. However, mechanical stress significantly increased osmotic fragility values of fresh PRBCs, especially after 6 h of simulation.

Erythrocytes may deform under a wide range of mechanical stresses and LORCA is capable of measuring the deformation, which is usually presented as the maximum elongation index (EI_max_) and half maximum elongation index (SS_1/2_). Boscarino et al.[15] exposed PRBCs to parachute descent and 12 h of simulated soldier patrol and found no shear stress-related differences. In our vigorous study of longer duration, EI_max_ and SS_1/2_ values showed no significant changes. Unsurprisingly, fresh erythrocyte deformability values were significantly better throughout the simulation.

The current study is not without limitations. Our primary concern was creating control groups that would not represent a real-life environment. Dividing each donor’s blood into two equal volumes and blood sampling would further decrease the blood volume. The duration of the experiment was set at 24 h due for the convenience of the simulator.

## Conclusion

Under the simulated conditions, we were unable to demonstrate the feasibility and safety of carrying PRBCs. Given the demonstrated benefits of transfusion in the PHP, our efforts shall not be hindered by the initial experience and new projects are underway.

## Figures and Tables

**Table 1:**
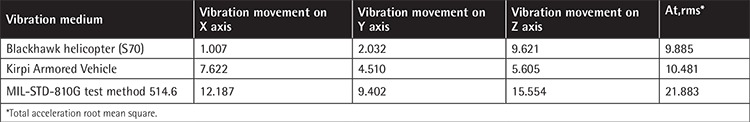
Acceleration (a) root mean square (rms) calculations in x, y, and z directions of three different vibration recordings.

**Table 2:**
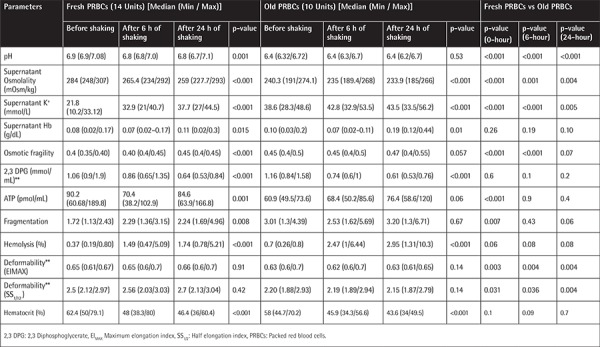
Effect of simulated Test Method 514.6 (MIL-STD-810G) shaking on biochemical and biomechanical values of fresh and old packed red blood cell stored in blood cooler box.

## References

[1] Bellamy RF, Maningas PA, Vayer JS (1986). Epidemiology of trauma: military experience. Ann Emerg Med.

[2] Bellamy RF (1984). The causes of death in conventional land warfare: implications for combat casualty care research. Mil Med.

[3] Champion HR, Holcomb JB, Lawnick MM, Kelliher T, Spott MA, Galarneau MR, Jenkins DH, West SA, Dye J, Wade CE, Eastridge BJ, Blackbourne LH, Shair EK (2010). Improved characterization of combat injury. J Trauma.

[4] Mabry RL, Holcomb JB, Baker AM, Cloonan CC, Uhorchak JM, Perkins DE, Canfield AJ, Hagmann JH (2000). United States Army Rangers in Somalia: an analysis of combat casualties on an urban battlefield. J Trauma.

[5] Holcomb JB, McMullin NR, Pearse L, Caruso J, Wade CE, Oetjen-Gerdes L, Champion HR, Lawnick M, Farr W, Rodriguez S, Butler FK (2007). Causes of death in U. S. Special Operations Forces in the global war on terrorism: 2001-2004. Ann Surg.

[6] Kelly JF, Ritenour AE, McLaughlin DF, Bagg KA, Apodaca AN, Mallak CT, Pearse L, Lawnick MM, Champion HR, Wade CE, Holcomb JB (2008). Injury severity and causes of death from Operation Iraqi Freedom and Operation Enduring Freedom: 2003- 2004 versus 2006. J Trauma.

[7] Eastridge BJ, Mabry RL, Seguin P, Cantrell J, Tops T, Uribe P, Mallett O, Zubko T, Oetjen-Gerdes L, Rasmussen TE, Butler FK, Kotwal RS, Holcomb JB, Wade C, Champion H, Lawnick M, Moores L, Blackbourne LH (2012). Death on the battlefield (2001-2011): Implications for future combat casualty care. J Trauma Acute Care Surg.

[8] Brown JB, Cohen MJ, Minei JP, Maier RV, West MA, Billiar TR, Peitzman AB, Moore EE, Cuschieri J, Sperry JL;, Inflammation and the Host Response to Injury Investigators (2015). Pretrauma center red blood cell transfusion is associated with reduced mortality and coagulopathy in severely injured patients with blunt trauma. Ann Surg.

[9] Çetinkaya RA, Yılmaz S, Eker İ, Ünlü A, Uyanık M, Tapan S, Pekoğlu A, Pekel A, Ertaş Z, Gürsel O, Muşabak UH, Yılmaz S, Avcı İY, Çetin AT, Eyigün CP (2015). In vitro efficacy of frozen erythrocytes: implementation on new blood stores to alleviate resource shortage (issue revisited). Turk J Med Sci.

[10] Parpart AK, Lorenz PB, Parpart ER, Gregg JT, Chase AM (1947). The osmotic resistance (fragility) of human red cells. J Clin Invest.

[11] Han V, Serrano K, Devine DV (2009). A comparative study of common techniques used to measure haemolysis in stored red cell concentrates. Vox Sang.

[12] Bhananker SM, Ramaiah R (2011). Trends in trauma transfusion. Int J Crit Illn Inj Sci.

[13] Malsby RF, Quesada J, Powel-Dunford N, Kinoshita R, Kurtz J, Gehlen W, Adams C, Martin D, Shackleford S (2013). Prehospital blood product transfusion by U. S. Army MEDEVAC during combat operations in Afghanistan: a process improvement initiative. Mil Med.

[14] Otani T, Oki K, Akino M, Tamura S, Naito Y, Homma C, Ikeda H, Sumita S (2012). Effects of helicopter transport on red blood cell components. Blood Transfus.

[15] Boscarino C, Tien H, Acker J, Callum J, Hansen AL, Engels P, Glassberg E, Nathens A (2014). Feasibility and transport of packed red blood cells into Special Forces operational conditions. J Trauma Acute Care Surg.

[16] Richieri GV, Mel HC (1985). Temperature effects on osmotic fragility and the erythrocyte membrane. Biochim Biophys Acta.

[17] Högman CF (1998). Preservation and preservation of red cells. Vox Sang.

[18] Spiess BD, Gillies BS, Chandler W, Verrier E (1995). Changes in transfusion therapy and reexploration rate after institution of a blood management program in cardiac surgical patients. J Cardiothoracic Vascular Anesthesia.

[19] Kinoshita A. Simulation of human erythrocyte metabolism. In: Madame Curie Bioscience Database [Internet]. Austin, Landes Bioscience, 2000-2013. Available online at.

[20] Zubair AC (2010). Clinical impact of blood storage lesions. Am J Hematol.

[21] Sowemimo-Coker SO (2002). Red blood cell hemolysis during processing. Transfus Med Rev.

[22] European Directorate for the Quality of Medicines (2015.). Principles of Blood Component Processing. Guide to the Preparation, Use and Quality Assurance of Blood Components, Recommendation No. R (95) 15. Strasbourg, EDQM,.

[23] Sağlık Hizmetleri Genel Müdürlüğü. Ulusal Kan ve Kan Bileşenleri Hazırlama, Kullanım ve Kalite Güvencesi Rehberi. Ankara, Sağlık Hizmetleri Genel Müdürlüğü, 2016. Available online at.

[24] Barshtein G, Gural A, Manny N, Zelig O, Yedgar S, Arbell D (2014). Storage-induced damage to red blood cell mechanical properties can be only partially reversed by rejuvenation. Transfus Med Hemother.

